# Sustainable Waste Management Strategies for Effective Energy Utilization in Oman: A Review

**DOI:** 10.3389/fbioe.2022.825728

**Published:** 2022-02-09

**Authors:** Kenneth E. Okedu, Hind F. Barghash, Husam A. Al Nadabi

**Affiliations:** ^1^ Department of Electrical and Communication Engineering, National University of Science and Technology, Muscat, Oman; ^2^ Department of Electrical and Electronic Engineering, Nisantasi University, Istanbul, Turkey; ^3^ Department of Environmental Engineering, German University of Technology, Muscat, Oman

**Keywords:** population growth, landfill, recycling, waste-to-energy, waste management, biohydrogen, hydrogen gas

## Abstract

Due to the adverse effect of energy production from traditional fossil fuels, the move toward renewable and sustainable energy has become imperative. The waste disposal sector offers hope toward environmental sustainability and potential to generate strong income for any economy. The region of the Gulf Cooperation Council (GCC) member countries in the Middle East generates the highest quantity of municipal waste per capita, compared to other countries globally. In the GCC region, waste is usually just dumped at different landfill stations. Effective waste management is imperative for environmental protection and sustainable development. This article presents a review of the sustainable waste management strategies in Oman, in line with the United Nations goal on environmental management of waste. The current waste management strategies in Oman were discussed, and various waste-to-energy (WTE) technologies were proposed. These proposed WTE strategies would help in effective waste management in Oman and also in increasing the power grid capability. The salient part of this article is an overview of the ability of using landfill leachate as feedstock to produce clean and green hydrogen gas using different photo-fermentation processes. The production of hydrogen using single photo-fermentation process using landfill leachate alone, with leachate and addition of inoculum such as sewage sludge, and with substrates such as sucrose, glucose, and landfill leachate was investigated by utilizing a batch bioreactor and anaerobic conditions at controlled temperature, different initial pH, and heat treatment temperature range. Furthermore, a prototype model for the optimization of the inoculum size and the substrate was proposed with 2-level factorial design using Design Expert. The data gathering for this study was carried out by investigating different regions in Oman having the lowest and highest waste produce, the average annual waste for the different governorates, and the total amount of waste for five consecutive years (2015–2020), for the various engineered landfills. The cost of managing waste in the different governorates of Oman was presented, considering the average annual waste produced, in various regions of the country for the duration of the study. Furthermore, some challenges and opportunities in carrying out effective waste management in Oman were addressed.

## Introduction

The amount of waste production is on the rise daily due to industrial activities, economic development, and population growth. Waste sources could be classified as domestic, commercial, industrial, municipal, and agricultural (Ilic´ and Nikolic, 2016). With the rapid development of industrialization and urbanization, urban sustainability has been challenged by the ever-worsening environmental problems and calls for a smart strategy to monitor their dynamics across different regions and countries. Recently, the attention toward environmental sustainability is increasing as one of the key elements for sustainable development, in order to achieve the United Nations Sustainable Development Goals. Gulf Cooperation Council (GCC) member countries (Saudi Arabia, Oman, the UAE, Kuwait, and Qatar) are considered major consumers of natural resources, which results in a larger number of emissions (Umar and Egbu, 2018).

According to previous studies in the literature, the production of municipal solid waste (MSW) may reach 2.6 billion tons per year by 2025, due to population increase, urbanization, industrialization, and economic development. The expansion of population in the GCC region has created economic growth and industrialization, which consequently promotes the production of MSW (Di Leo and Salvia, 2017a; Yousuf, 2018). A robust strategy to address waste management is therefore very important (Natália et al., 2017).

The waste-to-energy (WTE) process involves production of energy from combustion of non-recyclable residual waste in the form of electricity or steam. This strategy helps in clean energy production, mitigation of gases, and climate change effects because methane is not produced, unlike in landfills (Silva et al., 2017; Yang et al., 2017). The modern facilities used in WTE are designed to regain the value in waste after recycling by recovering clean energy, and they have air pollution control devices that help in decreasing the emissions.

The recent bio-based economy directing toward renewable energy development in the literature resulted in an overwhelming compulsion to identify new environmentally friendly processes for biomass conversion. In Antonis (2020), a holistic approach was employed in processing, developing, implementing, monitoring, and improving a strategy at a local and a central level, in the framework of waste management. The authors of the article proposed a methodology that is useful to develop and apply the scheme in household waste prevention, material reuse, and waste reduce in solid waste management. Similar studies were carried out in Antoniou and Zorpas (2019) and Aprile and Fiorillo (2019), for quality protocol development to define the end-of-waste method for tire pyrolysis oil in the framework of circular economy strategy and intrinsic incentives in household waste recycling, respectively. Combustion, gasification, and pyrolysis have been identified as promising thermochemical conversion techniques for energy and biofuel production in Simona et al. (2019). The authors of this article reported that many shortcomings need to be solved for developing their applications at large scale, one such is the influence of inorganics on char gasification reactivity. The potential of thermal plasma gasification of olive pomace charcoal was investigated in Tamošiūnas et al. (2017), combustion characteristics and kinetics of torrefied olive pomace in Guizani et al. (2016), and utilization of bottom ash as catalyst in biomass steam gasification for hydrogen and syngas production in Herman et al. (2016).

Food waste management is an extremely vital social and environmental issue. An idea of circular economy strategy in food waste management for the optimization of energy production via anaerobic digestion was presented in Pantelitsa et al. (2019). In this article, the authors presented results that support diverting food waste fraction of MSW to anaerobic digestion processes which forms part of the topology actions for achieving the objectives for waste and landfills. In Abeliotis et al. (2015), the implications of food waste generation on climate change were studied, while the environmental comparison of landfilling and incineration of MSW that accounts for waste diversion was reported in Assamoi and Lawryshyn (2012).

Globally, the ways to manage waste differ from one country to another. Approximately 14% of the raw materials used by German industries are recovered waste (Nellesa et al., 2015), thus reducing environmental impacts and contributing a share of about 20% based on the German Kyoto agreement on reducing climate emissions. Patrick Wager in 2007 (Patrick, 2007) proposed environmentally sustainable waste management policy in Switzerland. The proposed guidelines helped in waste treatment and reduced disposal; however, it created some challenges for the Swiss waste management system.

Recently, in the GCC region, the management of waste has been prioritized as a means of curtailing environmental pollution and proffering alternative solutions to power generation. According to Amna Shibeika, sustainable development is imperative for proper waste management (Amna et al., 2018). In the United Arab Emirates (UAE), about 4.55 million tons of waste were produced in the construction and demolition industry in the year 2016, indicating 47% of waste generated, compared to 34% in 2015. It was reported that 66% of these wastes were dumped in landfills. Furthermore, in a recent survey carried out in the UAE, about 96.5% of the participants agreed that lack of awareness and culture led to more waste production in the country (Amna et al., 2019). The generated waste in Saudi Arabia is regulated by the Local Affairs and Ministry of Municipalities by collecting and dumbing waste in open landfills or by combustion. However, disposing waste without treatment has huge environmental concerns because plastic waste that is recycled in Saudi Arabia is only 15–20% (Muzammil et al., 2016). In Bahrain, a study was carried out by Mohammed Saleh AL Ansari (Mohammed, 2012), on solid waste management. The study shows that in the last thirty years, there is a substantial growth of waste production in commercial, industrial, residential, institutional, construction, and demolition areas. The study also shows that waste management in Bahrain is faced with growth of population, fast industrialization, and lack of legislation with no enforcement mechanisms. Currently, MSW management in Bahrain is via waste collection and disposal at landfills at Al Manama, which serves the five governorates of Bahrain.

Umar in Umar (2020); Umar (2021a); Umar (2021b) investigated waste management and its use for energy production in Oman and other GCC countries. In Umar (2020), a framework was proposed for reducing greenhouse gas (GHG) emissions from MSW in Oman. In that study, the author proposed an attempt to develop frameworks that considered landfilling, composting, and recycling of MSW, by employing a quantitative research method to determine the composition of the MSW in Oman. In another study (Umar, 2021a), different frameworks used for estimating the GHG emissions from MSW in Oman was carried out. In that study, frameworks that considered landfilling, composting, and recycling of MSW were developed and comparison of the emissions of these frameworks was analyzed. It was concluded that the framework with the landfilling and composting process for the organic waste usually has an increase in emissions compared to the conventional landfill practice. A study involving qualitative and quantitative research methods was used to evaluate the MSW generation and emissions, electricity consumption and emissions, and public participation in waste segregation, in order to estimate the mitigation in emission considering WTE plant in Oman (Umar, 2021b). The results of the study showed that the current emissions from fossil fuels could meet certain electricity requirements that would help Oman improve its sustainability performance in energy, climate change, waste reduction, and economic growth. However, a more promising and clean energy production technique could be the production of hydrogen gas from waste in Oman.

The ability of using landfill leachate as feedstock to produce clean and green hydrogen gas considering different photo-fermentation processes was investigated (Barghash et al., 2021). The production of hydrogen using single photo-fermentation process using landfill leachate alone, with leachate and addition of inoculum such as sewage sludge, and with substrate such as sucrose and glucose and landfill leachate was investigated by utilizing a batch bioreactor and anaerobic conditions at a controlled temperature of 37 ± 1°C, different initial pH of 6, 6.5, and 7.2, and heat treatment at temperature range of 60–81°C in Barghash et al. (2021). All investigations were conducted at the light intensity range of 4,000–6,000 Lux with different mixing rates for shaking culture of 100, 200, and 400 rpm. And the hydraulic retention time used was 48 h. It has been observed that bio-hydrogen can be produced using landfill leachate alone, with the addition of inoculums/substrates such as sewage sludge, sucrose, and glucose.

In light of the aforementioned finding, this article presents a review of the sustainable waste management strategies for effective energy utilization in Oman, considering different regions of waste production in the country and the current ways of managing waste. WTE technologies that are envisaged in Oman for effective waste management and increase in the power grid capability were presented. The option of the WTE strategy could further be employed for smart grid operations for sustainable development in Oman. Since hydrogen gas is seen as a promising energy in the future, a cost-effective way of managing waste in Oman could be the production of green hydrogen gas from waste. This article presents an overview of the various strategies employed in producing hydrogen gas by biochemical processes and proposed an optimum scheme as a prototype that could be expanded for energy utilization. The cost of managing waste in the different governorates was presented for a period of 5 years (2015–2020), based on the average annual waste produced, and the amount of waste in the various engineered landfills. The huge cost of managing waste presently in Oman could be mitigated by using the waste-to-hydrogen strategy discussed in this article. Some obstacles that may occur in managing waste in Oman and some ways to go forward were also highlighted.

## Population and Waste Production in Oman

The amount of waste generated increases with expansion of cities and population increase. The World Bank issued a report in 2018 to highlight how the fast growth of cities, the economic development, and the population growth will make a significant contribution to increasing the amount of waste by 70% in the next 30 years, rising to 3.40 billion tons yearly. This is more than twice of the population growth by 2050 (Worldometer, 2020).

**FIGURE 1:**
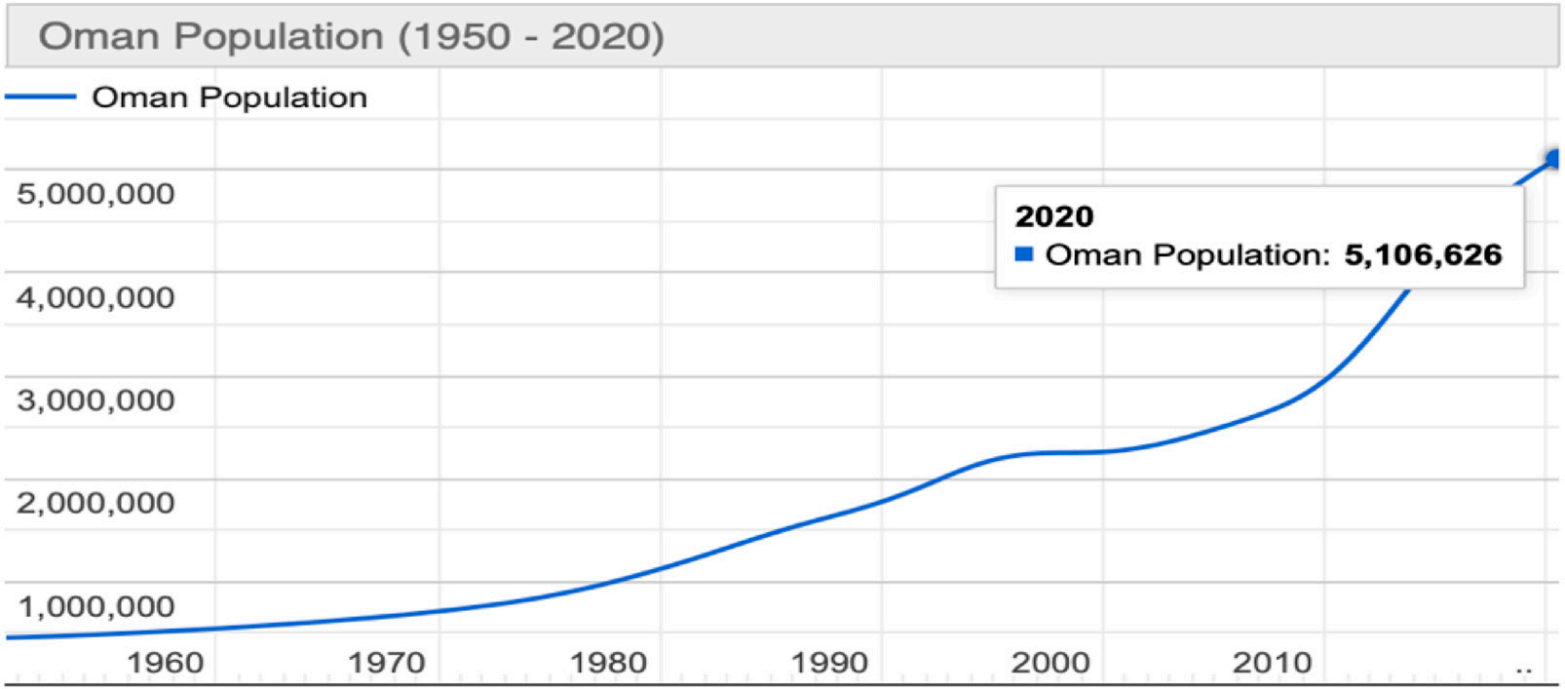
Population of Oman (1960–2020) (Be’ah, 2020).

Over the past decades, Oman has experienced a sudden change in the total population, with a population of around 723,881 in 1970 to a population of around 5,147,840 in 2020 (Be’ah, 2020), as shown in Figure 1. Approximately, 29% of the overall population live in Muscat (the capital city of the Sultanate of Oman). With a total land area of 3,500 km^2^, Muscat only comprises 1.1% of the total area of Oman.

Oman Environmental Service Holding Company (Be’ah) is responsible for solid waste management in the Sultanate of Oman. The major ways of moving the country forward in waste management is by establishing the necessary infrastructures, restructuring the municipal waste collection services, and enhancing the public consciousness of waste management. In the last 6 years, Oman had only one engineered landfill located in Muscat and utilized open dumpsites in the inland areas. For a long time, Oman was striving to launch 16 engineered landfills, 65 waste transfer stations, and four waste treatment plants in various parts of the country, especially in Muscat and Salalah. Today, over 380 dumpsites throughout Oman are switched to 11 engineering landfills (The Research Council of Oman, 2019).

In the region of Al Multaqa, located in Al Amerat city, there exists a landfill that is the first engineered sanitary landfill in Oman. The first cell started operation in 2011, and the life span of the first year was estimated at the end of 2014. The landfill site spreads across an area of 9.6 hectares, and it involves 5 cells with an overall capacity of 10 million m^3^ of solid waste and distributed over an area of 9.6 hectares. There are 16 shafts in every cell to take charge of leachate (polluted water).

Many countries developed advanced technologies for waste disposal by transitioning from landfilling and burning waste to establishing strategic projects that are built on scientific foundations for recycling, reducing the quantity of waste, protecting natural resources, preserving the environment, and creating new jobs. A survey carried out for Oman household waste indicates that the rate of household waste production in Oman is 1.5 million tons in 2012, and it is expected to increase to 1.89 million tons by 2030. This is equivalent to a rate of 1.2 kg per person per day (Be’ah, 2016). Consequently, there are needs to tackle waste management by using effective solutions and by switching this environmental issue into an economic resource by implementations of scientific approaches and schemes; otherwise, the problem will continue to linger on.

## Regional Waste Distribution in Oman

**FIGURE 2:**
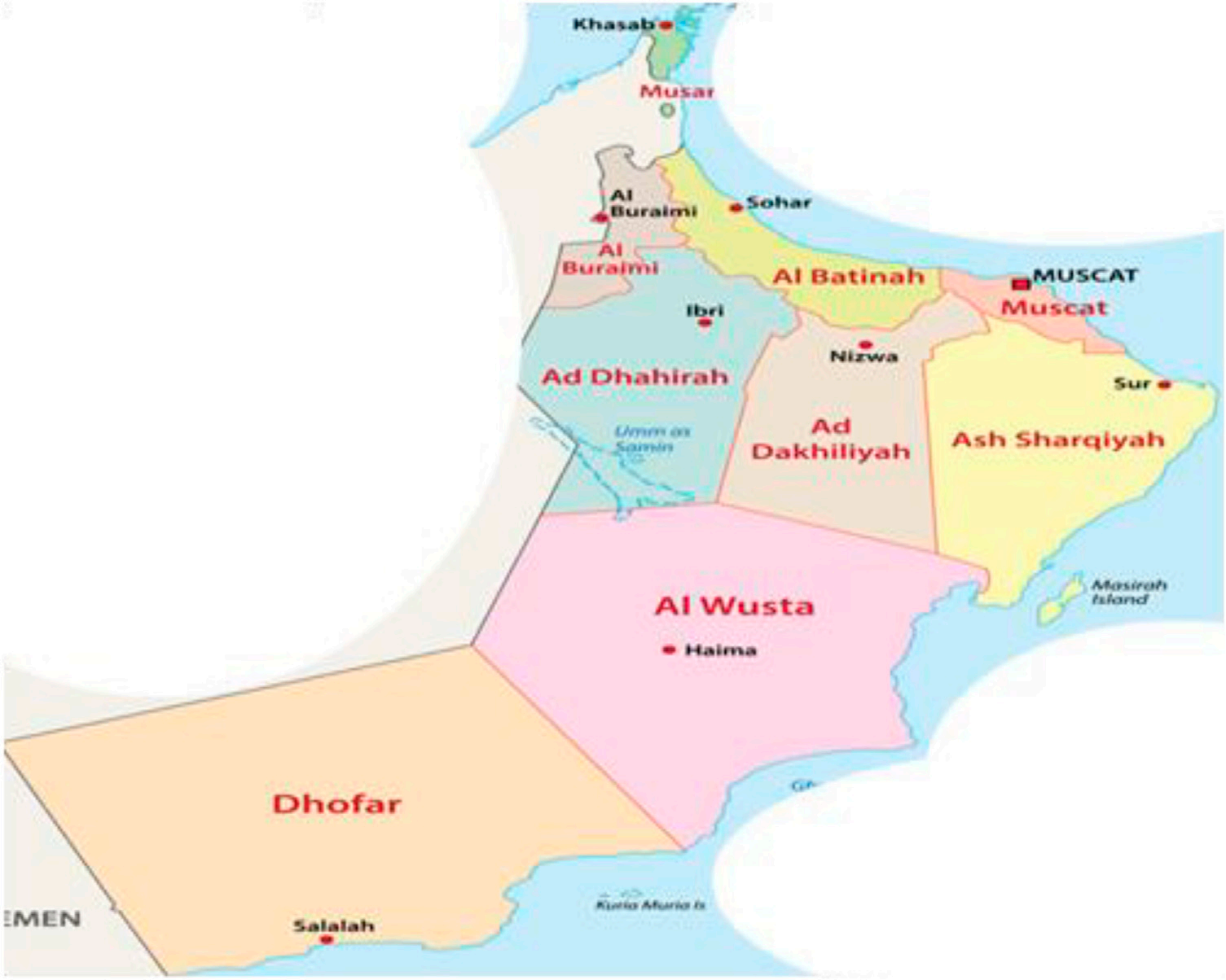
Waste distribution in Oman (Mohammed, 2015; Ithraa, 2016).

Figure 2 shows the map of Oman with regions having high and low waste based on Mohammed (2015). From Figure 2, it can be observed that Muscat, North Al Batinah, and Dhofar are the regions with high waste, while Musandam and Al Wusta are regions with low waste in Oman (Ithraa, 2016).

**FIGURE 3:**
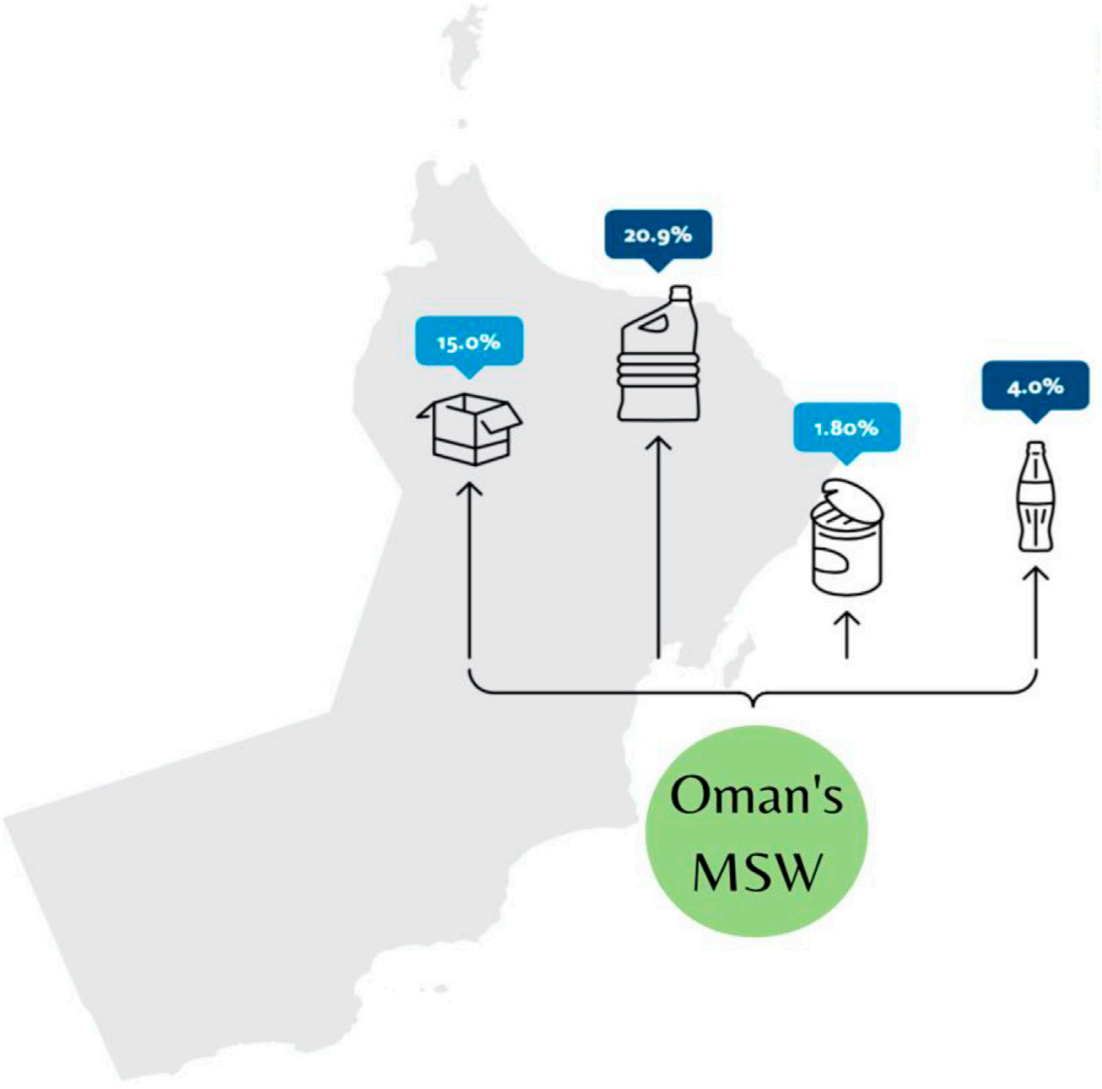
Percentages of recyclables in Oman.

The MSW of Oman was branded with a very large percentage of recyclables of mainly paper and cardboard (15%), 20.9% of plastics, 1.8% of metals, and 4% of glass, as shown in Figure 3 (Be’ah, 2019). The majority of MSW in Oman is transported to approximately 350 dumpsites (authorized and unauthorized) for disposal. The waste operations across the governorates of Oman are discussed as follows (Beigl et al., 2008).

**FIGURE 4:**
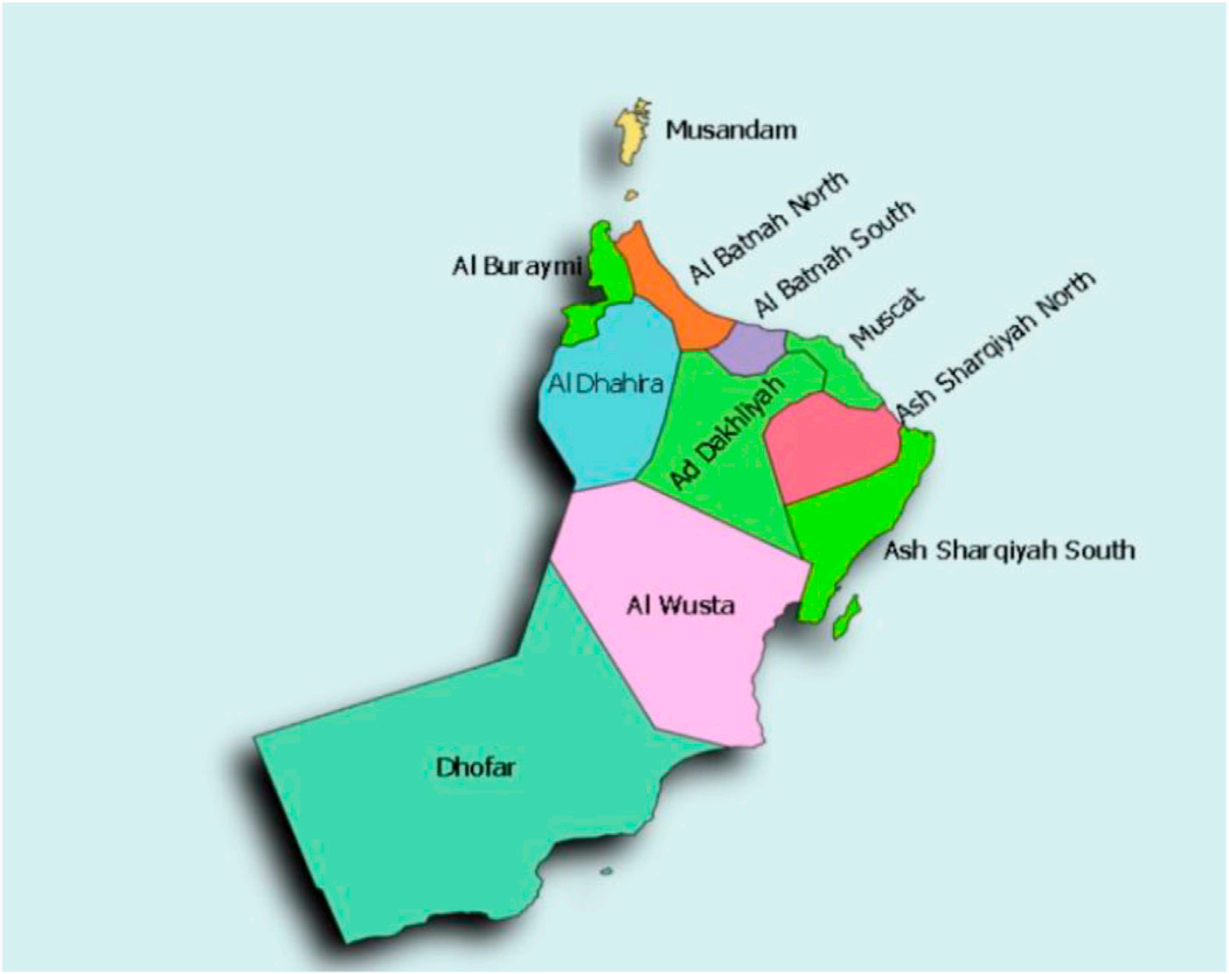
Regional waste distribution in Oman.

In 2015, MSW operations were initiated in South Al Sharqiyah governorate, as shown in Figure 4, covering the whole governorate along with Wilayat Muhot in Al Wusta. An average of 281 tons of waste was daily produced from these facilities in 2019. Along with over 111 collection trucks utilized for waste management operations, up to 17,797 bins were established in South Al Batinah since 2016, as shown in Figure 4. The average amount of waste generated in this governorate reached 497 tons per day. This governorate has about 22,153 bin operations. Al Dakhiliyah’s average daily waste production reached 445 tons/day, as shown in Figure 4. In Dhofar, 17,898 bins were situated across the governorate in 2017. The waste production of Dhofar reached an amount of 474 tons\day in Figure 4. The Al Dhahirah governorate has an average daily waste generation of 166 tons in Figure 4. In the Buraimi governorate, an average daily waste generation of 143 tons in Figure 4 was achieved in 2017. The north Al Batinah governorate has an average daily waste generation of 842 tons, as shown in Figure 4. Musandam’s governorate waste production reached 56 tons/day in 2019 in Figure 4. The operations of Muscat governorate are divided into two: Muscat 1 (part of Bausher, Qurum, Al Amerat, Quriyat, Muttrah) and Muscat 2 (Wilayt A’Seeb and part of Bausher). Muscat has an average waste daily production of 2,016 tons, as shown in Figure 4. Al Wusta’s governorate waste production reached 54 tons/day in 2019, as shown in Figure 4, while North Al Sharqiya governorate in 2019 produced an amount of 160 tons/day, as shown in Figure 4.

## Existing Waste Management Schemes in Oman

**FIGURE 5:**
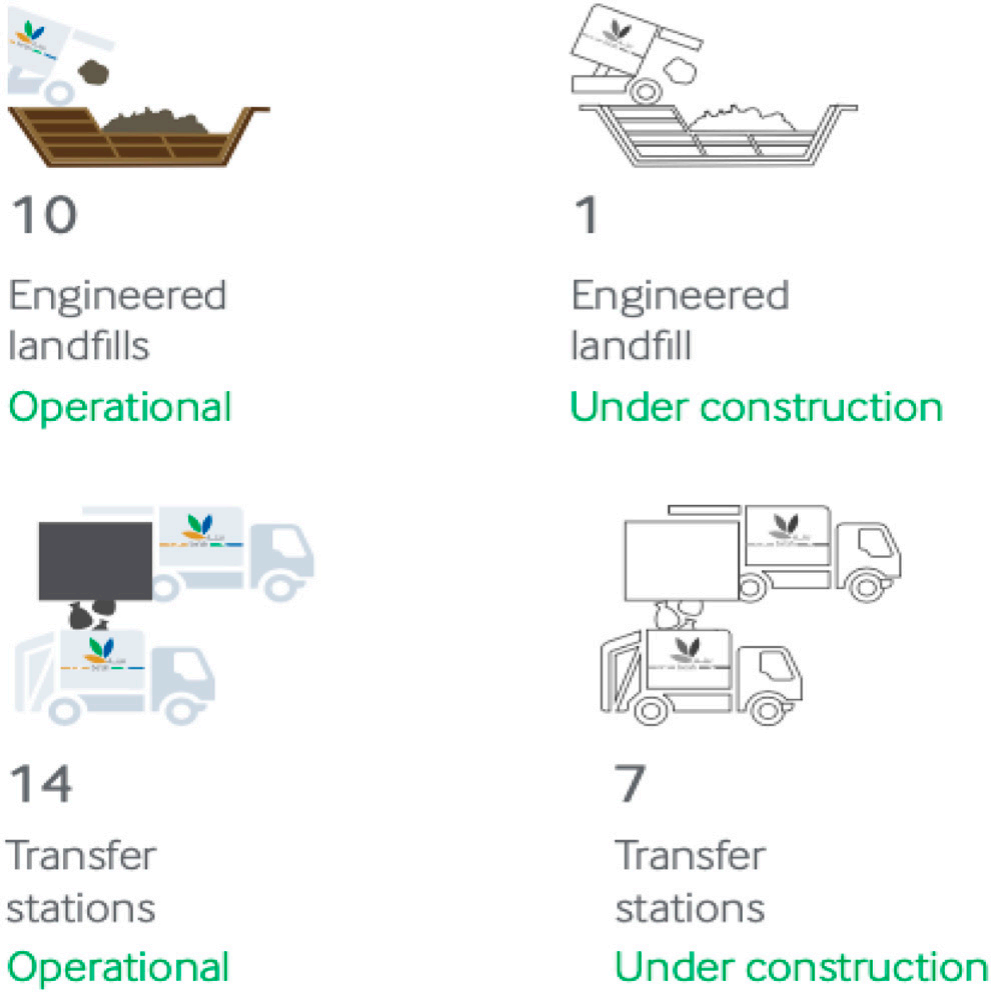
Current municipal solid waste infrastructure status in Oman.

There are 11 engineered landfills, about 18–125 waste transfer stations, and bulky item drop-off centers in the Sultanate of Oman, as shown in Figure 5. An engineered landfill is a well-designed pit, where layers of solid waste are positioned, compressed, and covered for final disposal. It is specially designed and developed to reduce pollution and health hazards, while transfer stations within the country are hubs where waste is gathered and classified before final disposal. These centers act as staging area where waste may be gathered and stacked before being forwarded into greater trucks to carry it to landfills. To guarantee smooth and swift operations, all the transfer stations are equipped with weigh bridges, prime movers, and hook lifts (Patrick, 2007).

**FIGURE 6:**
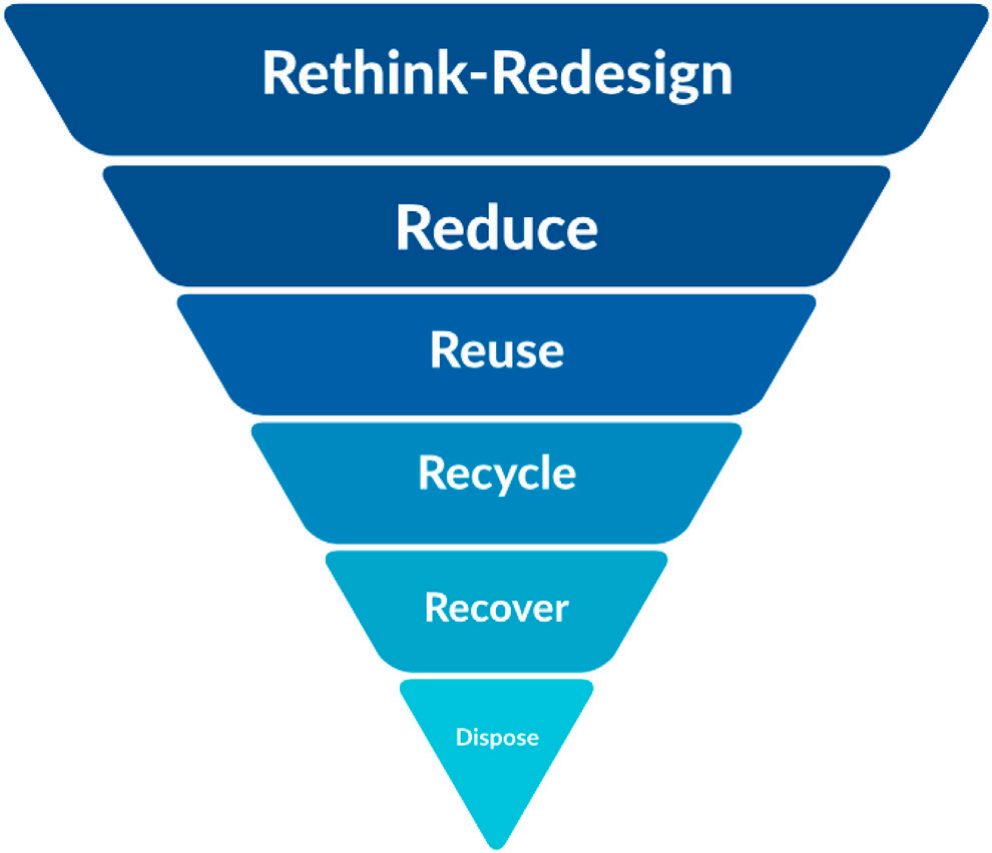
Ideal waste management hierarchy in Oman.

The main purpose of the waste management system is to minimize the negative environmental impact of waste and its adverse health effects. To fulfill this objective, it is vital to manage waste production, encourage the reuse of waste, support biological recovery and recycling, create awareness of using non-recyclable waste, and guarantee that treatment and disposal of waste have no negative effects. The waste management acts in Oman include the following four stages: waste disposal, minimal recycling, reuse, and reduce. Figure 6 shows the ideal waste management scheme employed in Oman, where a huge portion is allotted to rethinking, reduce, reuse, recycle, recovery and disposal. The main 4Rs in waste management in Figure 6 would help in waste management strategy in Oman, for a long-term MSW diversion methodology, and attain 80% of diversion rate by 2030. The detailed description of the 4Rs is given as follows.

**FIGURE 7:**
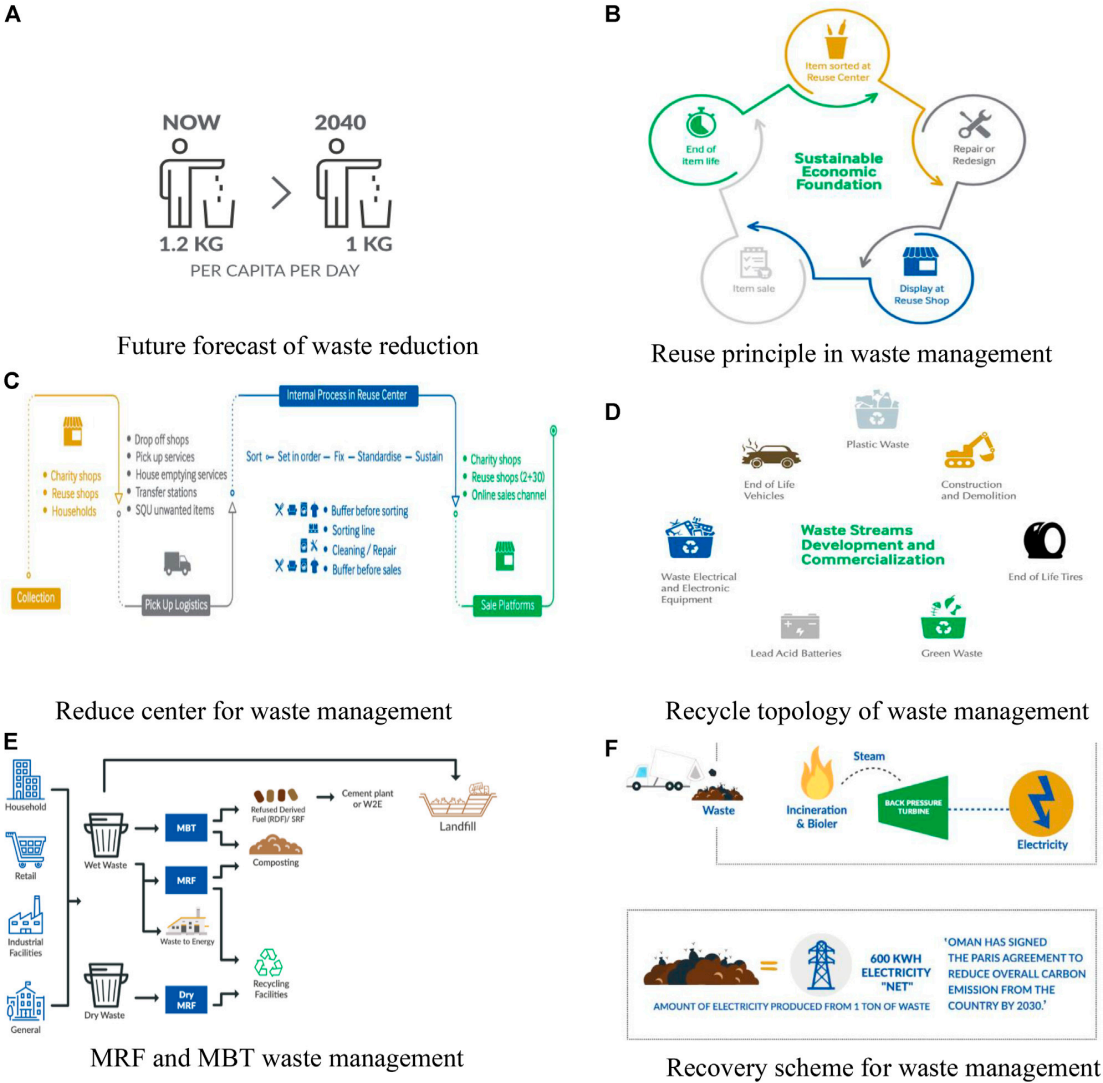
Existing waste management schemes in Oman. **(A)** Future forecast of waste reduction, **(B)** reuse principle in waste management, **(C)** reduce center for waste management, **(D)** recycle topology of waste management, **(E)** MRF and MBT waste management, and **(F)** recovery scheme for waste management.

Waste reduction is also known as source reduction, and it is the process of using less material and energy to minimize waste production. Thus, a less amount of waste will be treated and disposed. The purpose of this strategy is to reduce waste to less than 1 kg per capita per day in Oman, based on its vision 2040 sustainable development goals, *via* introducing the benefits of this approach in schools and by environmental awareness raising in societies. The current per capita per day waste production is 1.2 kg, as shown in Figure 7A. Reuse is the second preferred waste management approach, and it simply means giving the amount of waste generated a second life, instead of disposal, that is, using it over and over again. Figure 7B shows the principle of reuse in waste management. Be’ah and Sultan Qaboos University (SQU) in Oman have joined efforts to establish a reuse center at SQU, as shown in Figure 7C. The aim of this center is to contribute to the predisposal of waste diversion, *via* accepting and selling the reusable items from the donations offered by the Omani communities, public schools, charity organizations, and commercial institutions. Recycling is the procedure of gathering and processing materials to create a new product by adjusting its physical properties and manufacturing a brand new product. Figure 7D shows the recycle topology of waste management in Oman. Material recovery facilities (MRFs) and mechanical biological treatment (MBT) plants are sorting plants which support plans in the diversion of MSW from landfills, and in the reduction of disposal. Figure 7E shows the MRF and MBT waste management scheme employed in Oman. Recovery is identified as the diversion of waste materials from the waste stream via any waste management operation, as shown in Figure 7F. This procedure results in a specific product that has either economic potentials or ecological benefits (Di Leo and Salvia, 2017b). However, there are various alternatives to assist energy requirements of Oman by the recovery of energy from unrecyclable wastes. Energy-to-waste (ETW) is one of the energy recovery operations where energy is produced in the form of electricity from heat as a consequence of waste incineration. This technique could help deal with 3,800 tons of MSW per day. The treated waste will generate steam, and as a result, electricity will be produced.

## WTE Technology in Oman

WTE technologies are used to process waste into extract energy. The amount of energy that can be extracted depends on the type of waste, the efficiency of the plant, and the technique or the route utilized for energy recovery. Incineration is the combustion of organic substances contained in waste materials by using high temperature (Pincetel, 2010; Lim et al., 2014; Zaman and Swapan, 2016; Gehrmann et al., 2017), and this is one of the energy production methods. The generated heat may either be immediately used for heating purposes or be employed in the production of electrical energy. Mostly, incineration is applied to municipal solid waste, medical waste, and hazardous waste. There are various types of incinerators such as rotary kiln, fluidized bed, liquid injection, catalytic combustion, multiple hearths, waste gas flare, moving grate, and direct flame (Yang et al., 2017).

The moving grate and fluidized bed incinerators are most commonly used in the Omani industries. When applying moving grate incinerators, it is not necessary to sort waste or to remove any calorific value waste which can be converted into refuse-derived fuel (RDF) or solid-recovered fuel (SRF). However, pretreatment may be necessary in order to eliminate bulky substances. Under good conditions, it has been reported that the ideal calorific value for this technology can range from 7 kJ\kg to 14 kJ\kg. Generally, moving grate incinerators are preferred incinerators because they help attain the highest diversion from landfills, can treat up to 6,000 tons per day, and decrease the amount of waste on landfills. Unlike moving grate incinerators, sorting is essential in fluidized bed incineration to assure the high-value target of the caloric value of the waste, with less moisture contents. This sorted waste is classified as RDF or SRF, and it has been proven that the quality of SRF is greater than the quality of RDF. Moreover, this technology is more expensive because of two main reasons: first, it requires a precise boiler design, and second, the pretreatment of waste is expensive. Many WTE plants in Europe use this technology due to the fact that it is commercially and technically sustainable.

Currently in Oman, the reuse, recycle, and recovery method of waste is employed. In order to meet these goals, a reuse institute center at SQU to support prediversion of reusable materials, supporting source reduction and promoting the reuse mindset of society was proposed. There are also a number of projects to allow and enhance waste recycling like composting, polyethylene terephthalate (PET) recycling, diversion of fish waste, water recycling, scrap metal collection, and trade. Development of biogas and WTE plants is part of the strategy to improve waste recovery. It is noteworthy to mention that all the projects mentioned before are futuristic plans, and there are hopes to achieve them by 2025.

**FIGURE 8:**
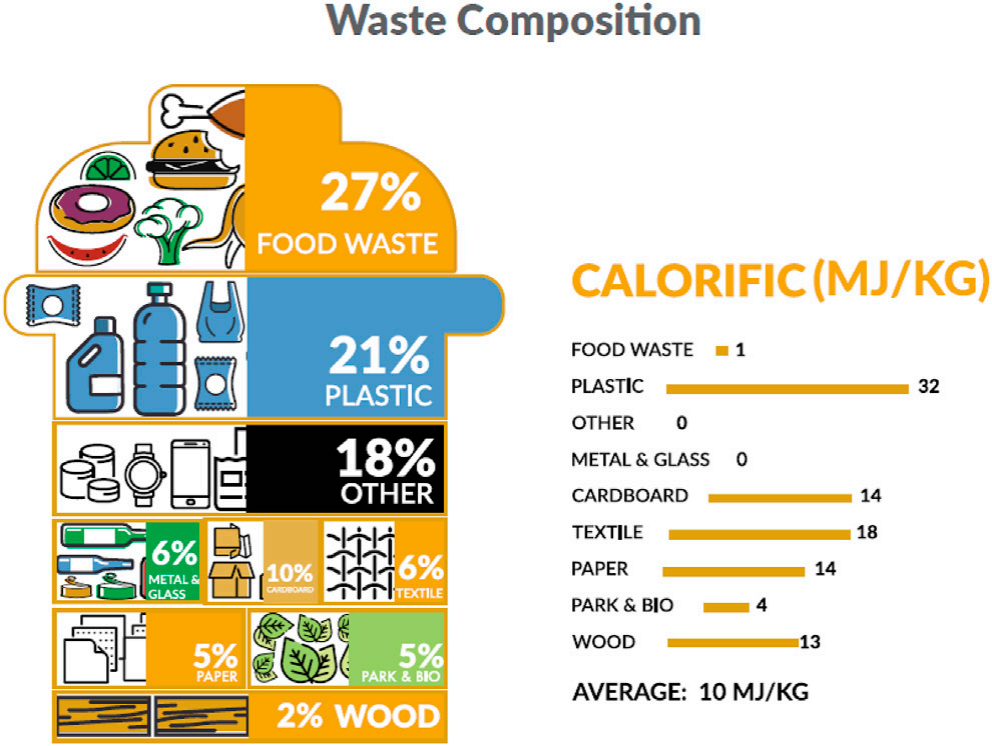
Calorific value of waste composition in Oman (Beigl et al., 2008).

**FIGURE 9:**
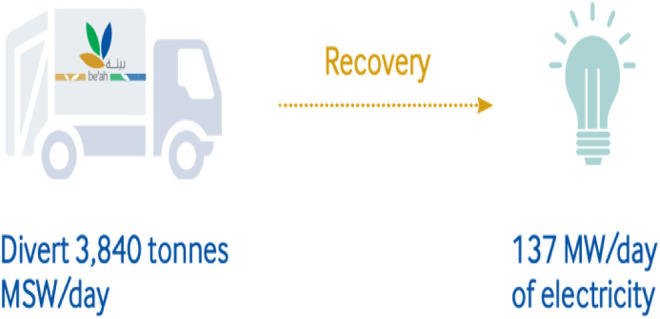
WTE plant in Barka, Oman.

**FIGURE 10:**
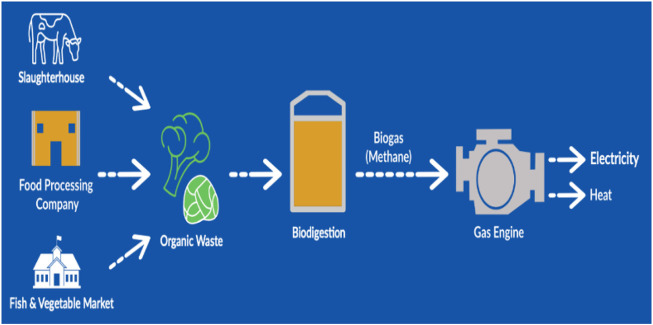
Organic waste diversion in Oman.

Figure 8 shows the calorific value of waste composites in Oman and the energy that could be extracted from these composites. From the figure, more calorific values of waste could be extracted from plastics, textiles, and cardboards, while a least calorific value would be obtained from metals, glasses, and food waste, respectively. Some proposed waste to energy techniques in Oman are discussed as follows. The WTE plant in Barka, as shown in Figure 9, is to divert about 3,840 tons of MSW daily, generating a net 137 MW\day of electricity that will be linked to the main power grid. The purpose of the organic waste diversion strategy is to employ organic waste in the production of biogas. Organic wastes like sludge, municipal waste, agricultural (green) waste, or food waste are considered as renewable sources of energy. Figure 10 shows the organic waste diversion strategy in Oman.

The aforementioned WTE proposed topologies would help in effective waste management in Oman and also in increasing the power grid capability. This strategy could be further employed for smart grid operations for sustainable development. However, since WTE could be the best option for the Oman waste management system, the previous proposed WTE options in Oman have limitations compared to the fully integrated waste management facilities in neighboring countries like Qatar. Therefore, the opportunities in fully integrated waste management facilities could be explored by Oman as discussed in the following subsection.

### Opportunities of Fully Integrated Waste Management Facilities in Oman

An environmentally sustainable path for waste disposal is WTE technology, in addition to clean energy production. The per capita waste generation in most GCC countries exceeds the global average of 1.2 kg per day with Kuwait being the highest in the region with 5.7 kg per day and Oman 0.7 kg per day (Jambeck Research Group Report, 2015; Marmore Mena Intelligence, 2018). The concern for WTE technology had not taken place in the GCC not until the past few years.

Globally, WTE technologies based on thermal energy conversion is widely preferred and accounted for approximately 88.2% of total market revenue. In Europe, WTE technologies accounts for 47.6% of the total market (Enerdata, IRENA, 2020). However, for developing countries, the major shortcoming in implementing WTE technology is the high cost. Consequently, the option of low-cost landfilling is widely preferred in these countries. Qatar has taken a bold step in implementing WTE technology in the GCC region.

Currently, there are no fully integrated waste management facilities in Oman, compared to the neighboring country like Qatar that is already employing this technology. In 2007, the government of Qatar awarded a contract to design and build the world’s largest fully integrated WTE plant in Doha (Government of Qatar, 2007). The integrated waste management facilities would help in reducing the bulk size of mixed MSW and recover useful resources substantially. This would definitely mitigate landfilling of waste to a large extent, consequently improving the useable life and extensions of landfills in Oman.

Generally, the fully integrated waste management facility is developed in phases, with the initial phase having a treatment capacity of 3,000 tons each day. An advanced incineration treatment technology is adopted along with mechanical sorting demonstration scale and several recycling schemes for recovery of useful resources from the original mixed MSW. These advanced technologies would mitigate pollutant emissions from incineration effectively, with double the amount of waste because of the advanced control technologies employed. Besides, another major benefit of using the topology of advanced incineration plants is that it will not lead to adverse health impacts since it complies with stringent emission standards (Environmental Protection Department, 2021). The fully integrated waste management facility will also have better air quality monitoring stations to generate objective data on local air quality for smooth operation and to ensure that the surrounding environment is not affected. A prototype of the fully integrated waste management facility used in Qatar could be extended to Oman. However, in this article, bio-hydrogen gas production based on the photo-fermentation process will be discussed as a future technology for effective waste management and energy utilization in Oman in the subsequent section.

## Overview of Waste to Hydrogen Gas Production in Oman Based on Photo-Fermentation Processes

There are four bio-hydrogen strategies that could be employed for the production of hydrogen gas from using landfill leachate that may be obtained from engineering landfills in Oman, considering different photo-fermentation processes. These four strategies are production of hydrogen using heat-treated leachate alone (strategy 1), production of hydrogen using leachate and heat-treated sewage sludge and sucrose (strategy 2), production of hydrogen using heat-treated leachate and glucose in fermenter (strategy 3), and hydrogen production using leachate and glucose as substrates (strategy 4).

**FIGURE 11:**
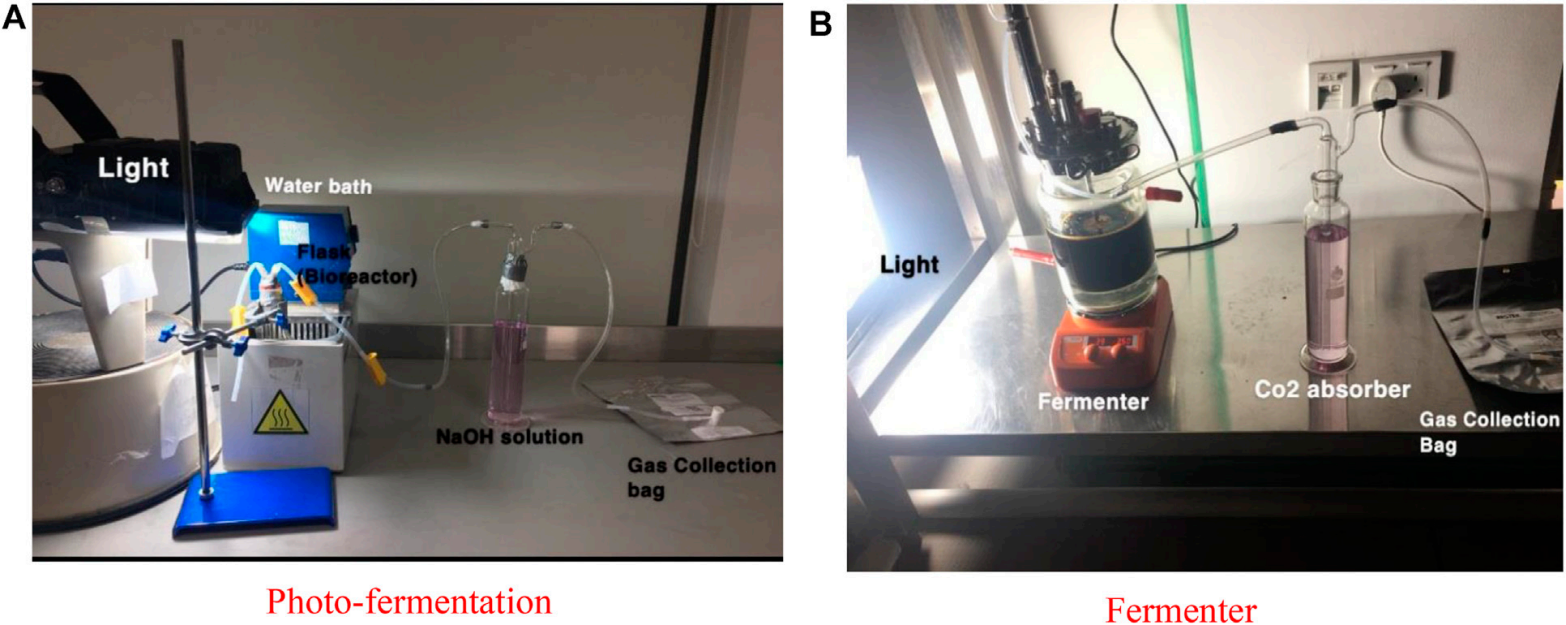
Experimental model of the study. **(A)** Photo-fermentation, **(B)** fermenter.

Figure 11 shows the experimental model used for this study. Figures 11A and B demonstrate the experimental setup for the photo-fermentation process and the fermenter. The fermenter has 2,000 ml capacity which works as a batch reactor, LED light with 150 W, a 300-ml gas washing bottle, 3-L gas sampling bag from RESTEK 2295, pipes, nitric acid HNO_3_, 98% sodium carbonate Na_2_CO_3_, and plastic clips. The fermenter is working with a stirrer at optimum rpm of 100 (Barghash et al., 2021). The bioreactor is connected to a washing bottle filled with 1 M NaOH solution and works as a CO_2_ absorber. The other pipe which is 4.8 mm thick is connected to the gas sampling bag, where other gases are collected and stored for further analysis. Also, the LED light position is opposite to that of the fermenter and adjusted at optimum illumination of 5,000–6,000 Lux.

The single photo-fermentation process was conducted using fermenter with 1 L working volume. Landfill leachate was used as a substrate, and sewage sludge was added to the batch reactor as inoculum. The sludge was sieved using a 1-mm mesh screen. In order to reduce the activities of the methane-producing bacteria, the sludge was heated at 80 ℃ for 30 min in a water bath. Afterward, the heat-treated sewage sludge was let to cool to room temperature and then mixed with leachate. The leachate was heated at 75 ℃ for 15 min. After the heat treatment, pH was adjusted using HNO_3_ and 1 M NaOH at 6.0. Then N_2_ (99%) is sparged for 15 min to the bioreactor. The working volume is 1 L, and the percentage of the added inoculum was optimized using Design Expert. Also, the substrate of sucrose/glucose was added to the batch reactor. Furthermore, nitrogen (99%) was sparged for 15 min, and the process was conducted at a controlled temperature of 37 ℃ . The optimization of the inoculum size and the substrate was done using 2-level factorial Design Expert.

**FIGURE 12:**
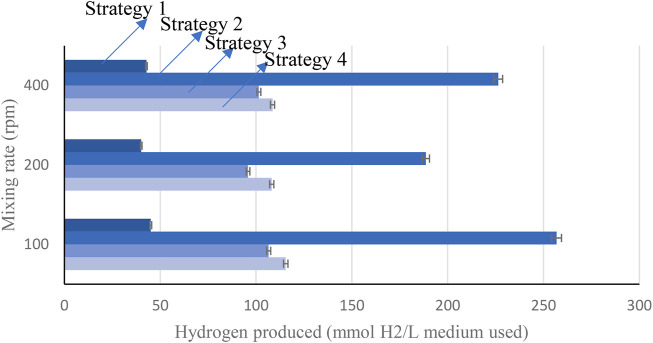
Hydrogen gas produced.

**FIGURE 13:**
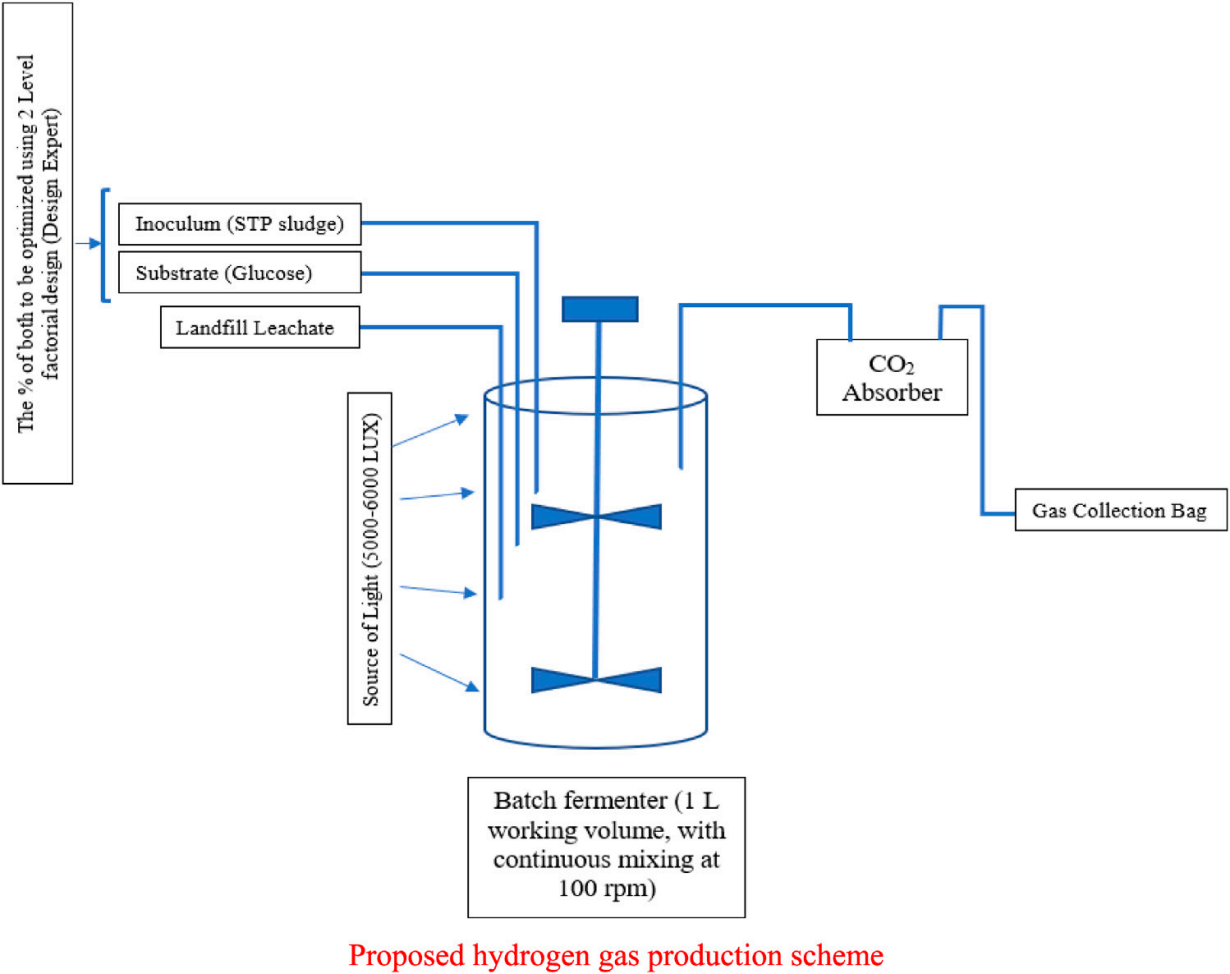
Proposed hydrogen gas production scheme.

Higher production of hydrogen was achieved while mixing leachate with sewage sludge, resulting in the production of 5,754 ml H2/L medium, in a batch reactor with continuous mixing at 100 rpm with a medium pH scale of 6.0 at an optimum light intensity range of 5,000–6,000 Lux. Higher production was also observed using more leachate with mixing of landfill leachate with glucose, resulting in the production of 2,589.2 ml H_2_/L, in a batch reactor with continuous mixing at 400 rpm with a medium pH scale of 6.0 at an optimum light intensity range of 5,000–6,000 Lux, as demonstrated in the presented results of Figure 12. The results presented in Figure 12 reflect that optimum hydrogen would be produced from the engineering landfills of Oman by using the topology of leachate and heat-treated sewage sludge and sucrose. The proposed hydrogen production scheme in Figure 13 would be investigated in the near future, as part of this study.

## Waste Management Cost in Oman Using the Existing Schemes

**FIGURE 14:**
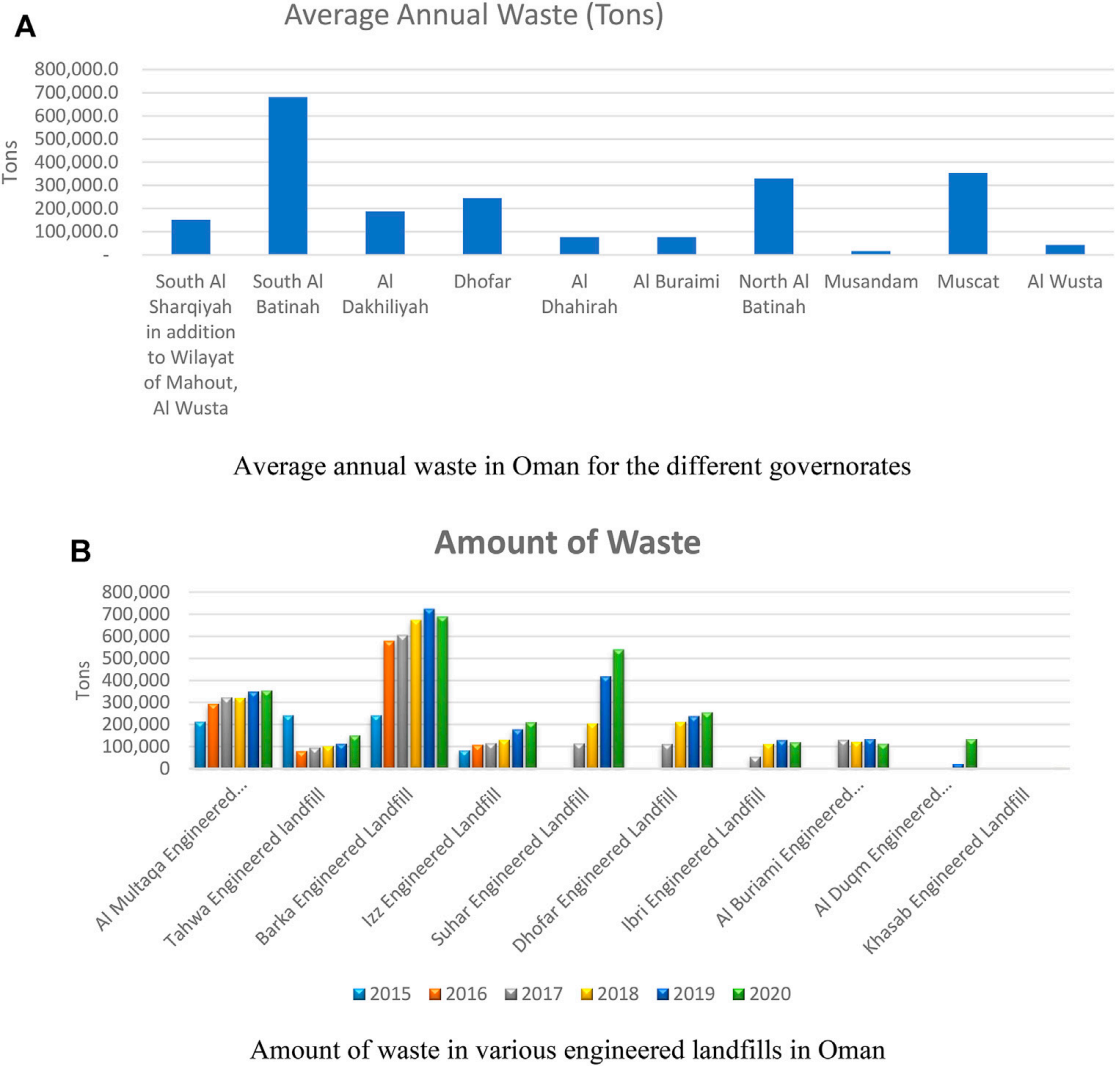
Amount of waste in Oman. **(A)** Average annual waste in Oman for the different governorates and **(B)** amount of waste in various engineered landfills in Oman.

The total investment of Oman for basic MSW infrastructure is estimated to be approximately USD 150 million. The waste-to-energy projects has around USD 750 total investments, and the investments in the industrial waste infrastructure will reach more than USD 150 million (Be’ah, 2016). The evaluation of the average annual waste, amount of waste, and the cost of managing waste in Oman are presented in this section. This cost could be reduced by using the proposed waste to hydrogen waste management strategy in *Overview of Waste to Hydrogen Gas Production in Oman Based on Photo-Fermentation Processes*. Figure 14A shows the annual waste produced in the 11 governorates of Oman. Although 10 governorates are shown in Figure 14A, South and North Sharqiyah were merged. From Figure 14A, South Al Batinah governorate has the highest annual waste of about 680,000 tons. This is because this region has the highest population in Oman, and there are much farming activities resulting in a huge amount of waste products. Muscat governorate which is the capital is the second governorate with huge amounts of waste products. This is because of the influx of foreigners, commercial, and industrial activities. North Al Batinah governorate has a slight drop in annual waste than Muscat governorate. The least amount of waste in the Sultanate is in Musandam governorate, with less than 20,000 tons of waste in a year. The amount of waste in Oman for five consecutive years (2015–2020) is shown in Figure 14B, for the various engineered landfills.

From Figure 14B, Barka, which is a city in the South Al Batinah governorate, has the highest amount of waste in the engineered landfills, based on Figure 14A. The Al Multaqa engineered landfill, located in the Muscat governorate, is having large amounts of waste after the Barka engineered landfill. The least amount of waste could be found in the Khasab engineering land fill which is located in Musandum, thus correlating with the data presented in Figure 14A.

**FIGURE 15:**
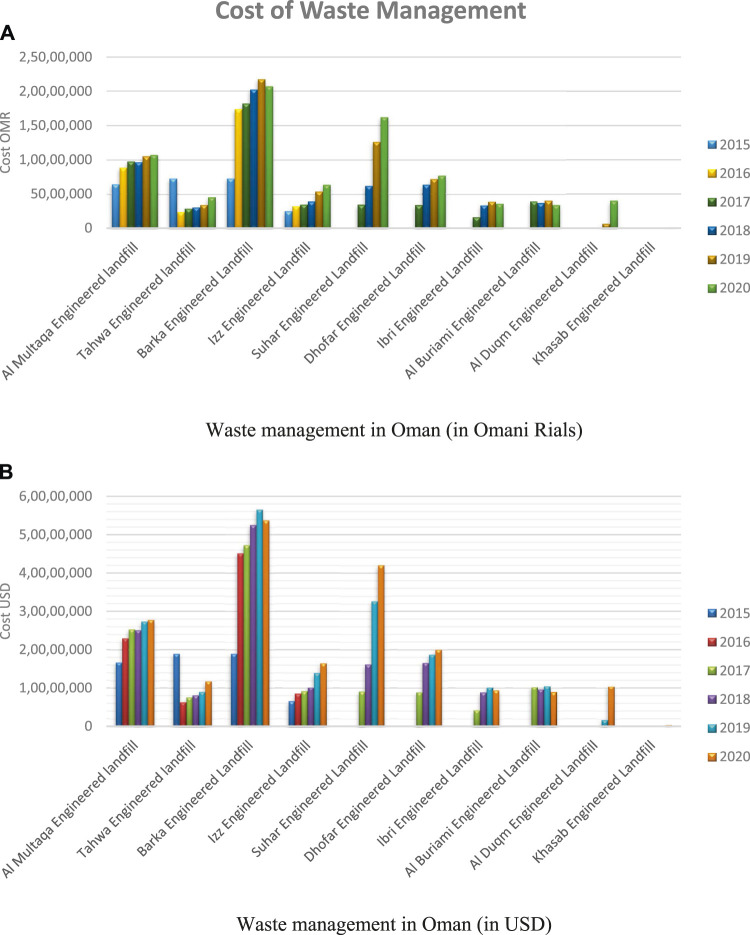
Cost of waste management in Oman. **(A)** Waste management in Oman (in Omani rials) and **(B)** waste management in Oman (in USD).

Figures 15A,B show the cost of managing waste in Oman in Omani rials and in USD, respectively, for five consecutive years. The cost of managing the Barka engineered waste is more as expected based on Figures 14A,B, respectively. This gradually increased from about 7 million Omani rials in 2015, to about 22 million Omani rials in 2019, which was the peak, before decreasing in the year 2020, to about 21 million Omani rials. Also, from Figure 15A, the Al Multaqa engineered landfill, located in Muscat governorate, is more expensive to manage after the Barka engineered landfill. Little or no cost is required to manage the Khasab engineering landfill which is located in Musandum, thus correlating with the data presented in Figures 14A,B, respectively.

In general, in 2020, for waste management in Oman, an average of 100 million Omani rials per year is needed (Be’ah, 2020). This is quite expensive; thus, it is imperative for the government of Oman and private sectors to look for alternative ways of reducing or managing waste in the Sultanate. One of such recent measures was the ban of plastic bags by the government on January 1st, 2021. Other measures are expected for effective control of waste management in the country like reuse of waste, biological recovery and recycling, waste material from waste stream, plastic waste control, rethinking, reduce, reuse, and recycle, recovery, disposal, and organic waste control. A preferred scheme would be the proposed waste to hydrogen proposed in this article.

## Challenges and Opportunities of Waste Management in Oman

Some of the challenges of waste management in Oman can be summarized as follows. There are inadequate strategic and no clear-cut master plan for waste management. The lack of specific laws and regulations is another major issue in the implementation of waste management. Besides, there are no integrated system and facilities for effective waste management. Due to lack of institutions in waste management, there are no reliable data and records for research purposes and effective planning. The absence of skilled and experienced waste management staff is another shortcoming in managing waste in Oman due to the nature of the job.

The opportunities of waste management in Oman are as follows. Effective waste management would lead to a healthier environment that is safe to live in. Since most of the bulk energy in the power grid of Oman is from fossil fuel and gas turbines, energy from waste could be used as an alternative source of renewable energy to augment the expensive traditional source of energy. Also, the energy obtained from waste could be used to power remote communities not connected to the power grid at a very low rate. Decreasing environmental pollution would increase the carbon credit of Oman, and the effect of climate change would be reduced. In addition, effective waste management would lead to development of some waste conversion machineries, power generation, and exchange of technologies that could contribute to the economic benefits of the country. Furthermore, jobs would be created in the operation of waste management machineries and disposal of waste in the country.

## Conclusion

This article presented a review of the sustainable waste management solutions in Oman. The different regions of Oman considering population and the amount of waste produced were investigated. The existing waste management schemes in Oman were highlighted. The strategies of waste management based on rethinking, reduce, reuse, recycle, recovery, and disposal were also discussed. Besides, some calorific value of waste composites in Oman and energy that could be extracted was highlighted. WTE and organic waste diversion schemes were reviewed in Oman as an alternative for effective waste management. However, these schemes have not been fully implemented. Currently, there are no fully integrated waste management facilities in Oman, compared to the neighboring country like Qatar that is already employing this technology. The technology of the integrated waste management facilities would help in reducing the bulk size of mixed MSW and recover useful resources substantially. Therefore, the government of Oman can key into such technology for more effective and sustainable waste management. The WTE topologies discussed in this article would help to effectively manage waste in Oman and also in increasing the power grid capability. This strategy could further be employed for smart grid operations for sustainable development.

The article also presented an overview of hydrogen gas production from engineering landfills in Oman, by photo-fermentation processes in a fermenter experimental setup. Optimum hydrogen was produced considering the conversion of waste from the engineering landfills of Oman, by using the strategy of leachate and heat-treated sewage sludge and sucrose. This prototype scheme could be expanded as a more effective way of waste management through biochemical process and the same time produce hydrogen gas for energy utilization in Oman. The cost of managing waste in the different governorates of Oman was presented, considering the average annual waste and the amount of waste in the different engineered landfills. Due to the high expenses incurred by the government of Oman and private sectors in managing waste in the country, it is paramount that alternative ways of reducing or managing waste in the Sultanate should be employed. Consequently, it is recommended to device new measures like the ban of plastics non-biodegradable bags and also the main 4Rs measures for effective control of waste management in the country. However, the overview of the waste to hydrogen gas strategy presented in this article would be a cost-effective scientific approach to reduce the current high cost of managing waste in Oman. Furthermore, some challenges and opportunities in carrying out effective waste management in Oman were also addressed in this article.

## Data Availability

The original contributions presented in the study are included in the article/Supplementary Material; further inquiries can be directed to the corresponding authors.
